# Total mutational load and clinical features as predictors of the metastatic status in lung adenocarcinoma and squamous cell carcinoma patients

**DOI:** 10.1186/s12967-022-03572-8

**Published:** 2022-08-18

**Authors:** Karen Y. Oróstica, Juan Saez-Hidalgo, Pamela R. de Santiago, Solange Rivas, Sebastian Contreras, Gonzalo Navarro, Juan A. Asenjo, Álvaro Olivera-Nappa, Ricardo Armisén

**Affiliations:** 1 Centre for Biotechnology and Bioengineering (CeBiB), Department of Chemical Engineering, Biotechnology and Materials, University of Chile, 8370456 Santiago, Chile; 2 Department of Computer Science, University of Chile, 8370459 Santiago, Chile; 3 Department of Cell and Molecular Biology, Faculty of Biological Sciences, Pontificia Universidad Católica de Chile, Santiago, Chile; 4 Department of Basic Clinical Oncology, Faculty of Medicine, University of Chile, Santiago, Chile; 5 Centro de Genética Y Genómica, Instituto de Ciencias E Innovación en Medicina, Facultad de Medicina Clínica Alemana, Universidad del Desarrollo, 7590943 Santiago, Chile; 6 Max Planck Institute for Dynamics and Self-Organization, Göttingen, Germany; 7 Present Address: Instituto de Investigación Interdisciplinaria, Vicerrectoría Académica, Universidad de Talca, 3460000 Talca, Chile

**Keywords:** Random Forest, Smoking, Clinical variables, Lung Adenocarcinoma (LUAD), Lung Squamous Cell Carcinoma (LSCC) and Metastasis

## Abstract

**Background:**

Recently, extensive cancer genomic studies have revealed mutational and clinical data of large cohorts of cancer patients. For example, the Pan-Lung Cancer 2016 dataset (part of The Cancer Genome Atlas project), summarises the mutational and clinical profiles of different subtypes of Lung Cancer (LC). Mutational and clinical signatures have been used independently for tumour typification and prediction of metastasis in LC patients. Is it then possible to achieve better typifications and predictions when combining both data streams?

**Methods:**

In a cohort of 1144 Lung Adenocarcinoma (LUAD) and Lung Squamous Cell Carcinoma (LSCC) patients, we studied the number of missense mutations (hereafter, the Total Mutational Load TML) and distribution of clinical variables, for different classes of patients. Using the TML and different sets of clinical variables (tumour stage, age, sex, smoking status, and packs of cigarettes smoked per year), we built Random Forest classification models that calculate the likelihood of developing metastasis.

**Results:**

We found that LC patients different in age, smoking status, and tumour type had significantly different mean TMLs. Although TML was an informative feature, its effect was secondary to the "tumour stage" feature. However, its contribution to the classification is not redundant with the latter; models trained using both TML and tumour stage performed better than models trained using only one of these variables. We found that models trained in the entire dataset (i.e., without using dimensionality reduction techniques) and without resampling achieved the highest performance, with an F1 score of 0.64 (95%CrI [0.62, 0.66]).

**Conclusions:**

Clinical variables and TML should be considered together when assessing the likelihood of LC patients progressing to metastatic states, as the information these encode is not redundant. Altogether, we provide new evidence of the need for comprehensive diagnostic tools for metastasis.

**Supplementary Information:**

The online version contains supplementary material available at 10.1186/s12967-022-03572-8.

## Background

Lung cancer (LC) is the most common cause of cancer-related mortality worldwide, responsible for more than 1.4 million deaths per year [1]. The two major subtypes of LC, lung adenocarcinoma (LUAD) and lung squamous cell carcinoma (LSCC), are classified as non-small cell lung cancers (NSCLC) [2]. Despite the common classification, these NSCLCs are likely to have drastically different clinical outcomes; LSCC and LUAD patients have an overall survival rate of 18% and 65%, respectively, when treated with tailored therapy [3, 4]. However, patients can receive tailored therapy only after typification, i.e., identifying what kind of LC they have. Despite advances in genomic characterization, associating genomic information (as, e.g., mutational profiles) with the clinical outcomes in NSCLCs remains an open challenge, given its complexity and heterogeneity [5].

Together with the mutational signatures characteristic of each LC subtype, certain clinical variables can help the typification of a tumour. For example, smoking has been recognised as the leading risk factor for LC, especially for the LUAD subtype [6, 7]. Specific genes are affected in these patients depending on whether they are smokers or not. For example, non-smoker LUAD patients typically present driver mutations EGFR, KRAS, TP53, and fusions in ROS, EML4-ALK, and RET genes [8]. On the other hand, smoker LUAD patients commonly have KRAS mutations [9]. The tumour mutational burden (TMB), defined as the total number of somatic mutations per coding area of a tumour genome, encodes some of the information above. As tumours with high TMB are likely to express more neoantigens that may sensitise them to immunotherapy [10, 11], TMB has been used as a predictor of immunotherapy response and effectiveness across various tumour types [12, 13]. Therefore, including the number of mutations could further characterise tumour progression in NSCLCs.

Recently, large-scale sequencing techniques have led to the accumulation of genomic information in cancer research. This comprehensive mapping of the mutational signatures of tumours has allowed researchers to use machine learning models to solve classification problems or predict relevant clinical outcomes. One of these clinical outcomes is whether a patient will develop metastasis, which is the leading cause of death in cancer patients [14]. Therefore, finding which factors (among clinical and genomic) are most informative in these models—and thus are better predictors for metastasis development—is crucial for identifying risks to develop metastasis in the early stages of cancer. Finally, this information would support medical practitioners adapt their therapeutic strategies when treating LC patients.

In this study, we put forward a new variable to quantify the accumulation of missense mutations in the whole exome: the Total Mutational Load (TML). Through the TML, we account for potential effects of the accumulation of missense mutations in metastasis development, as these may impair tumour-suppressing proteins or promote the development of proto-oncogenes, thus favouring cancer cells proliferation [15]. First, we studied the distribution of the TML and clinical variables across patients with different LCs and clinical categories. Then, using Random Forest (RF) machine learning models, we evaluated how informative the TML and other clinical variables (e.g., age, tumour stage, and smoking status) were to classify metastasis development in 1144 Pan-Lung Cancer samples. Finally, we compared different data preprocessing and processing alternatives to identify the one that produces the best-performing models. Altogether, we provide new insights on the factors that could allow an early identification of patients at risk of developing metastasis, and improve understanding of the relationship between genomics and clinical variables in NSCLC patients.

## Methods

### Dataset and data preprocessing

Clinical and mutational data from the Pan-Lung Cancer 2016 dataset was obtained from The Cancer Genome Atlas (TCGA) repository [16, 17]. This dataset contains 1144 LUAD and LSCC patients (“examples”, from a data-analytic perspective, Table 1). Protocols for patient recruitment, tumour sampling, pathological analysis, DNA extraction, and NGS library generation follow the ABSOLUTE methodology, are carefully described in [18]. In summary, patients and samples were obtained from multiple hospitals participating in TCGA. Sample processing and pathological assessment were done centrally at the TCGA Biospecimen Core Resource, following the strict TCGA protocol. A single kit was used to prepare all the NGS libraries, the Agilent SureSelect Human All Exon 50 Mb kit, followed by Illumina sequencing (paired-end). Finally, the bioinformatic analysis was standardised, and various sequencing quality controls were applied to avoid bias and batch effects.

**Table 1:** Distribution of clinical features in 1144 Pan-Lung Cancer samples

Clinical features	Entire cohort N = 1144	%
Metastasis class		
M0	731	63.9%
M1	22	1.9%
MX	210	18.4%
Cancer type		
Lung Adenocarcinoma	660	57.7%
Lung Squamous Cell Carcinoma	484	42.3%
Tumour stage		
Stage I	8	0.7%
Stage IA	246	21.5%
Stage IB	321	28.1%
Stage II	4	0.3%
Stage IIA	129	11.3%
Stage IIB	174	15.2%
Stage III	3	0.3%
Stage IIIA	155	13.5%
Stage IIIB	34	3.0%
Stage IV	8	3.3%
Age range		
< = 60 years	61	29.5%
> 60 years	146	70.5%
Gender		
Female	468	40.9%
Male	673	58.8%
N stage		
N0	624	54.5%
N1	221	19.3%
N2	113	9.9%
NX	16	1.4%
Smoking status		
Current reformed smoker for < or = 15 years	407	35.6%
Current reformed smoker for > 15 years	212	18.5%
Current reformed smoker, duration not specified	86	7.5%
Current smoker	271	23.7%
Lifelong non-smoker	111	9.7%

For the statistical analysis, we applied a filter to work only with those examples where all values for the clinical features we evaluated were reported (N = 948). Then, to predict metastasis status (M stage), we worked only with examples where this variable was reported (N = 728).

### Determination of total mutational load (TML)

We filtered mutational data to consider only missense mutations and created an m x n mutation count matrix, where m = 1144 and n = 17305, respectively, account for the number of patients (examples) and genes analysed. Each entry of the count matrix *V*_i,j_ indicates the number of missense mutations in the *j’th* gene observed in the *i’th* patient. Then, we used the *mutCountMatrix()* function from the Maftools R package [16] to obtain the number of missense mutations. As the number of genes was much larger than the number of examples, we filtered out genes where we observed a near-zero variance within the cohort, using the *VarianceThreshold()* function of the sci-kit learn python package [17] with a threshold of 0.05. We then computed the TML for each patient, given by Eq. 1:

1 $${TML}_{i}= {\sum }_{j}^{M}{V}_{i,j}$$

where TML: Total mutational load.

### Statistical analysis

#### Association between TML and clinical variables

For the statistical analysis, we considered patients’ TML and the following clinical variables: sex, tumour stage, age, M stage, number of cigarette packages smoked per year, and smoking history. Since TML is an over-dispersed count variable, we modelled it as a negative binomial random variable and used a negative binomial regression (NBR) explanatory model. Then, we fitted model parameters to the clinical variables mentioned above. Next, we applied a backward stepwise model selection over the NBR to determine the effect of each clinical variable on the TML. For this purpose, we used the *drop1()* function with a likelihood-ratio test (LRT), selecting predictors using a statistical significance of 0.05. Finally, we applied the *Student’s t-*test to determine whether the mean TML is equal across and within groups of patients filtered by clinical categories.

#### Reclassification of patients using the regional lymph node parameter

The M stage indicates whether cancer cells have spread from the primary tumour to other parts of the body. We defined three categories for this feature: M0 (cancer has not spread), M1 (cancer has spread), and MX (the pathologist could not determine whether cancer has spread). We reclassified patients into two groups: reclassified localised cancer (RM0) and reclassified advanced cancer (RM1). Each group of patients *P* results by combining the following disjoint categories: (Eq. 2).

2 $$\begin{aligned} P_{RM0} & = P_{M0} \cup P_{Mx, N0} \\ P_{RM1} & = P_{M1} \cup P_{Mx, N1} \cup P_{Mx, N2 } \cup P_{Mx, N3} \\ \end{aligned}$$

where New groups are defined as reclassified localised cancer (RM0) and reclassified advanced cancer (RM1) based on metastasis status and regional lymph nodes parameter (N).

The RM1 group was defined based on the regional lymph nodes parameter (N), which indicates whether cancer has spread to surrounding lymph nodes. N0 indicates no spreading to lymph nodes, while N1, N2, and N3 show that cancer has reached the lymph nodes in different degrees. Here, we hypothesise that cancer spread to lymph nodes is a precursor to developing metastasis. Consistently, it is unlikely that a patient in the N0 class could have any metastasis. Therefore, we used RM1 as a positive metastasis class and RM0 as a negative one for training and comparing our classification models.

### Random forest models

To identify the best predictors of metastasis status in patients with LUAD and LSCC, we built four Random Forest (RF) classification models. In these models, we use a combination of TML and clinical features to determine the metastasis status [19]. To assess which of these variables allowed a better prediction of the metastasis status, we used different combinations of TML and clinical variables as follows:

1. RF Model 1: MS ∼ Clinical variables + TML
2. RF Model 2: MS ∼ Clinical variables (excl. Tumour stage) + TML
3. RF Model 3: MS ∼ Clinical variables
4. RF Model 4: MS ∼ Clinical variables (excl. Tumour stage)

Each model was benchmarked as described in the next section.

### Benchmarking of the classification models

**Fig. 1:**
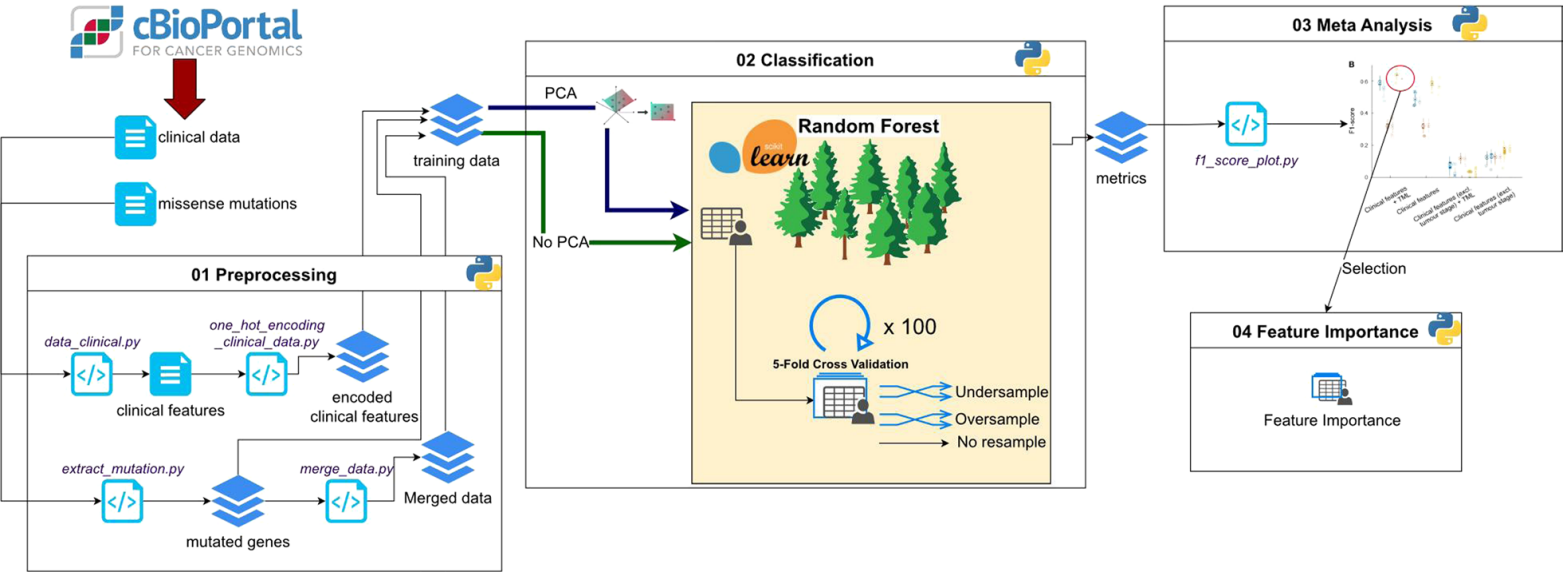
Benchmarking stages. The proposed benchmarking for model comparison has four main stages. First (stage 01), we preprocess the dataset and apply different classification and validation strategies to generate an input dataset. Second (stage 02), we train Random Forest models using different subsets of the input dataset, aiming to assess the relative importance of each data stream. In this stage, we also evaluate whether applying dimensionality reduction techniques (PCA) and different resampling schemes affects model performance. We repeat the experiment on 100 different partitions (training and validation) of the input dataset, obtaining performance distributions instead of single values. Third (stage 03), we analyse the performance distributions and error sources to assess which strategies perform better under each condition. Finally (stage 04), we selected the best model for the dataset studied and identified the feature that most contributed to the classification

Our model benchmarking analysis consisted of four stages (Fig. 1). In the preprocessing stage (01), we checked the data for consistency and encoded categorical variables (i.e., representing them numerically) using the One-Hot Encoding method [20]. We also standardised the TML around zero, subtracting the mean and dividing by the sample standard deviation. We then merged both datasets (standardised TML and encoded clinical variables) into the final input dataset. Before that, however, we generated different subsets to assess how informative each of the variables was. In an on–off basis, we studied the effects of i) including or not the tumour stage, and ii) including or not the TML (i.e., four possibilities).

Second, we performed a classification stage (02). Due to the high number of predictors (i.e., inputs to the classification model) in the input dataset for RF models 1 and 2, we performed a PCA to reduce its dimensionality. For this, after preprocessing, we apply a PCA to each input dataset and preserve only the 100 most informative principal components. These were the effective input datasets for the model evaluation, training, and assessment. Noteworthy, PCA is applied on the whole input dataset; If the calculation was performed independently on the training or validation sets, the resulting principal components would be biased to the data selection and the particular partition. Then, to prevent overfitting and class imbalance when splitting the dataset between training (70%) and validation (30%), we tested three different sampling methods: oversampling, undersampling, and no resampling [21]. We applied this to both datasets (with PCA and without PCA) and used the resulting training sets to generate our models. As the splitting between the training and validation datasets was random, the 70/30 partition was repeated 100 times, using different random seeds, thus generating 100 different models (per dataset and sampling method).

The Meta-analysis stage (03) consisted on using the validation dataset to obtain performance metrics (Accuracy, Recall, Precision and the F1 score) and assess the classification power of each of the generated models. Performance metrics were defined as follows:

$${\text{Accuracy = }}{{\left( {{\text{TP }} + {\text{ TN}}} \right)} \mathord{\left/ {\vphantom {{\left( {{\text{TP }} + {\text{ TN}}} \right)} {\left( {{\text{TP }} + {\text{ FP }} + {\text{ TN }} + {\text{ FN}}} \right)}}} \right. \kern-\nulldelimiterspace} {\left( {{\text{TP }} + {\text{ FP }} + {\text{ TN }} + {\text{ FN}}} \right)}}$$

$${\text{Recall = }}{{{\text{TP}}} \mathord{\left/ {\vphantom {{{\text{TP}}} {\left( {{\text{TP }} + {\text{ FN}}} \right)}}} \right. \kern-\nulldelimiterspace} {\left( {{\text{TP }} + {\text{ FN}}} \right)}}$$

$${\text{Precision = }}{{{\text{TP}}} \mathord{\left/ {\vphantom {{{\text{TP}}} {\left( {{\text{TP }} + {\text{ FP}}} \right)}}} \right. \kern-\nulldelimiterspace} {\left( {{\text{TP }}+{\text{ FP}}} \right)}}$$

$${\text{F1 = }}{{{\text{2}}*(\left( {{\text{Precision}}*{\text{Recall}}} \right)} \mathord{\left/ {\vphantom {{{\text{2}}*(\left( {{\text{Precision}}*{\text{Recall}}} \right)} {\left( {{\text{Precision}} + {\text{Recall}}} \right)}}} \right. \kern-\nulldelimiterspace} {\left( {{\text{Precision}} + {\text{Recall}}} \right)}}{\text{ }}$$

where: TP: True positive rate, TN: True negative rate,FP: False positive rate, FN: False negative rate.

As we had 100 different validation datasets, instead of a single value, we obtained a distribution of performance metrics and used them to assess classification performance and uncertainty. We compared the models generated using different datasets and sampling criteria, selecting those with the lowest false positive rate and the highest true positive rate. Finally, we assess which of the strategies for preprocessing (i.e., with or without PCA) and resampling (without, oversampling, or undersampling) produced the best-performing models, and rank the importance of the different features contributing to achieve the classification of metastatic status (04). The above is critical, as by identifying the most informative features in the classification of patients with known metastatic status, we can assess the risk of new patients developing metastasis.

## Results

### Relationship between the TML and clinical variables

**Fig. 2:**
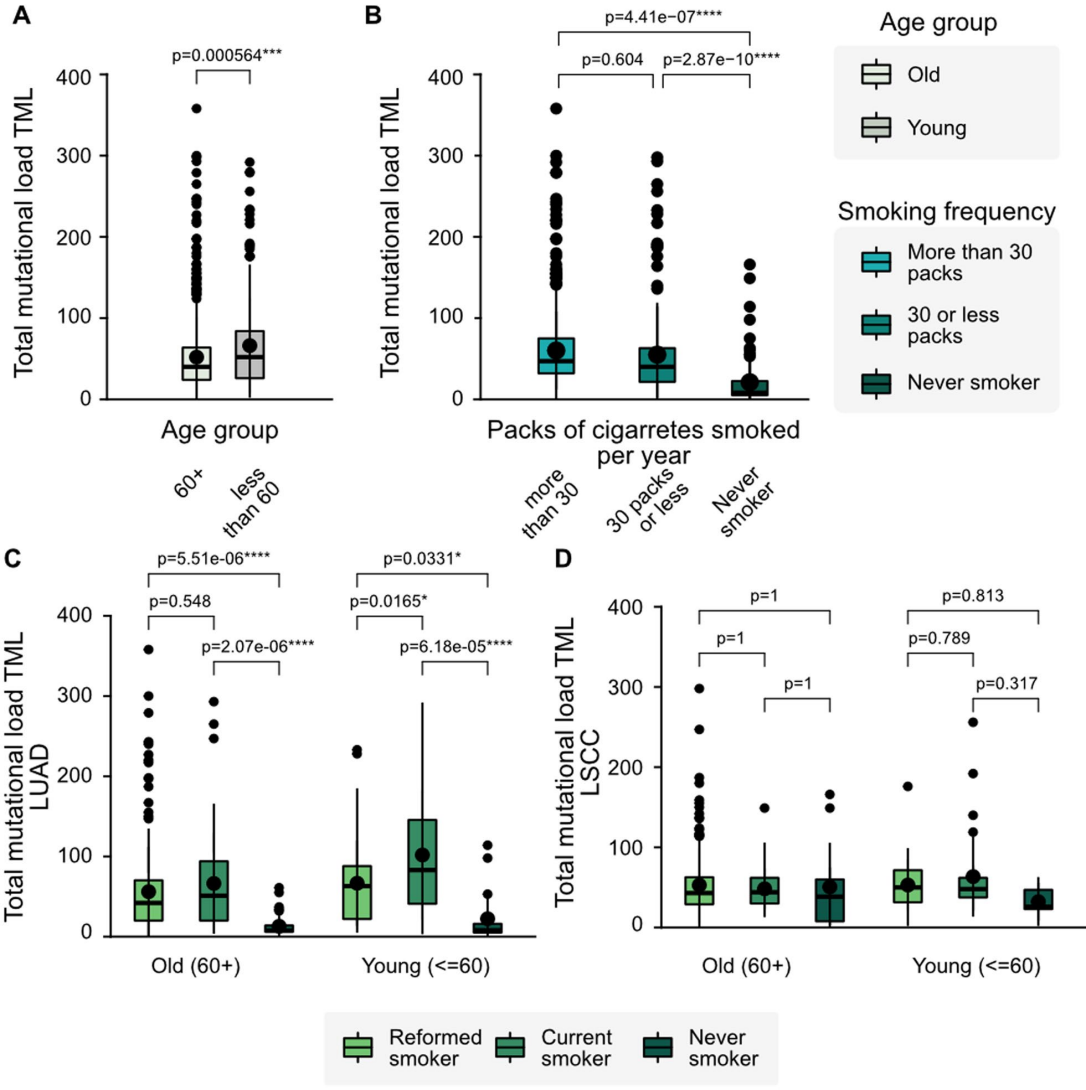
Age, smoking history, and cancer type affect the TML in all patients. (**A**) TML by age-range. > 60 years old, n = 713, ≦ 60 years old, n = 208. (**B**) TML by smoking history. Lifelong non-smoker, n = 83, ≦ 30 cigarette packs smoking, n = 235, > 30cigarrette packs, n = 491. (**C**, **D**) Statistically meaningful differences between the median of the TML distributions were found only for LUAD patients, hinting to LSCC having other origins than smoking. Current smokers have the highest TML for both age groups, and, within that group, young patients seem to have a higher TML than older patients. LUAD: lung adenocarcinoma; LSCC: lung squamous cell carcinoma. TML: Total Mutational Load; ***** p < 0.0001; ** p < 0.001; NS non-significant

**Fig. 3:**
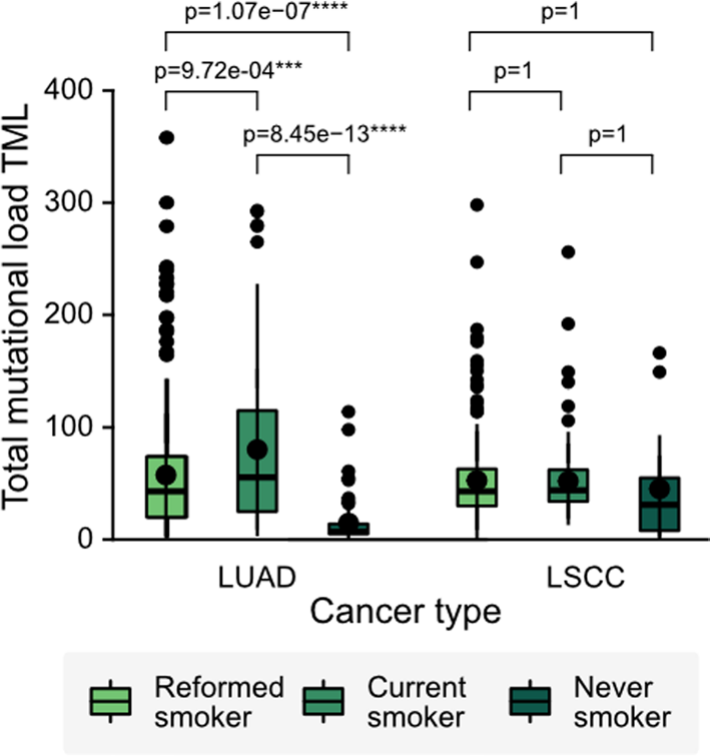
Smoking history affects the TML in LUAD but not in LSCC patients. LUAD patients: Current reformed smoker, n = 287, current smoker, n = 110, and lifelong non-smoker, n = 66. LSCC patients: Current reformed smoker, n = 321, current smoker, n = 124, and lifelong non-smoker, n = 17. TML: Total Mutational Load; LUAD: lung adenocarcinoma; LSCC: lung squamous cell carcinoma

Patients belonging to different categories of age, packs of cigarettes smoked per year, and cancer type had significantly different mean TMLs (cf. Figure 2 and Table 2). When we compared the TML of patients in different age ranges, we found younger LC patients (≦ 60 years old) to have higher TMLs compared to older patients (51.7 vs 66.0) (Fig. 2A). Also, smoker patients who have smoked more than 30 packs of cigarettes annually have, on average, a higher TML than those who have smoked less than 30 packs per year (respectively 60.1 and 55) and those that are lifelong non-smokers (21.0) (Fig. 2B). As is shown in Fig. 2, the TML is affected by cigarette consumption in LUAD, as lifelong non-smokers have a significantly lower TML than current and reformed smokers (Fig. 3).

**Table 2:** Association between clinical data and TML

Clinical features	LRT	P-value
Sex	0.2670	0.09
Tumour stage	5.5828	0.5512
Age range	5.6112	0.0010**
M stage	5.6824	0.4351
Cigarette packs per year	3.8632	0.0477740*
Smoking history	2.4781	0.3604040
Cancer type	9.4596	0.0008893***

^*^p < *0.1*

^**^p < *0.01*

^***^p < *0.001*

When we evaluate the mean TML by cancer subtype, considering the age ranges and types of smokers, we find that for LUAD patients, the mean TMLs were significantly different, both for those under and over 60 years independently. Also, the current smokers had the highest TML, while never-smokers had a lower mean TML. An interesting finding was that current young smokers have a higher mean TML than patients older than 60 years (Fig. 2C). However, for LSCC patients, we do not see these significant differences. The TML means are similar between age and types of smokers groups (Fig. 2D). Considering the cancer type, we found that the TML was higher in patients with LUAD than in patients with LSCC (57.8 vs 52.4) (Fig. 3). Interestingly, we found that cigarette consumption does not impact the TML in LSCC patients (Fig. 3).

### Reclassification of patients using cancer spread to lymph nodes

**Fig. 4:**
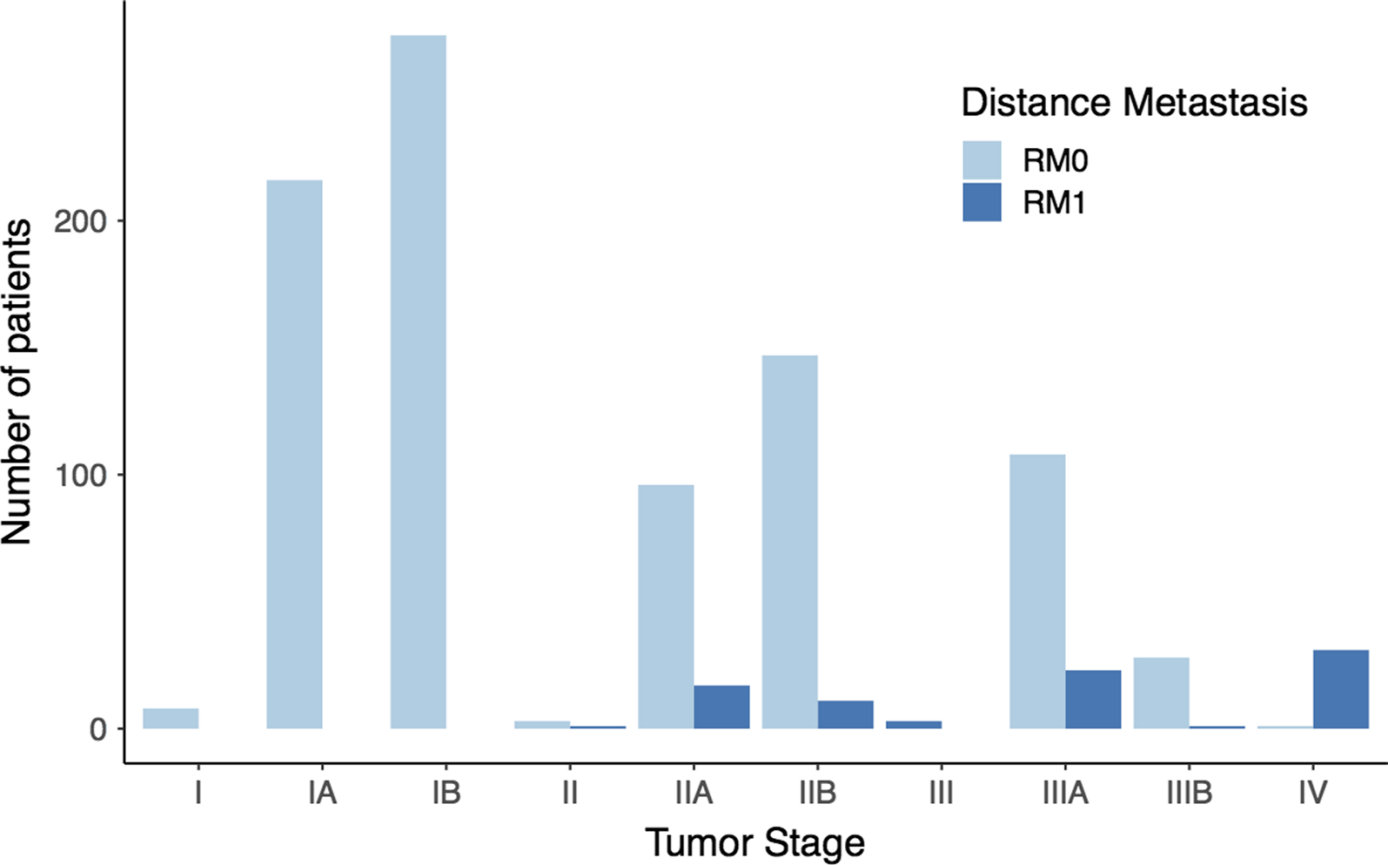
Reclassification of the metastatic status of Pan-Lung cancer patients using cancer spreading to lymph nodes. Distribution of RM0 and RM1 groups across tumour stages

As described in Methods, we reclassified the patients into two groups, RM0 (n = 880) and RM1 (n = 87), using the regional lymph nodes parameter (N), which indicates whether cancer has spread to surrounding lymph nodes. Figure 4 shows the results of the reclassification across cancer stages. The I, IA, and IB stages do not have patients classified as RM1, while IIA, IIB, III, IIIA, and IIIB stages have a low proportion of RM1 patients. Moreover, most patients in stage IV were recategorised to RM1, implying a specific relationship between these variables. Despite this reclassification that considers the spread of cancer to lymph nodes, there is still a clear class imbalance in the dataset.

### Classification of patients using RF models

**Fig. 5:**
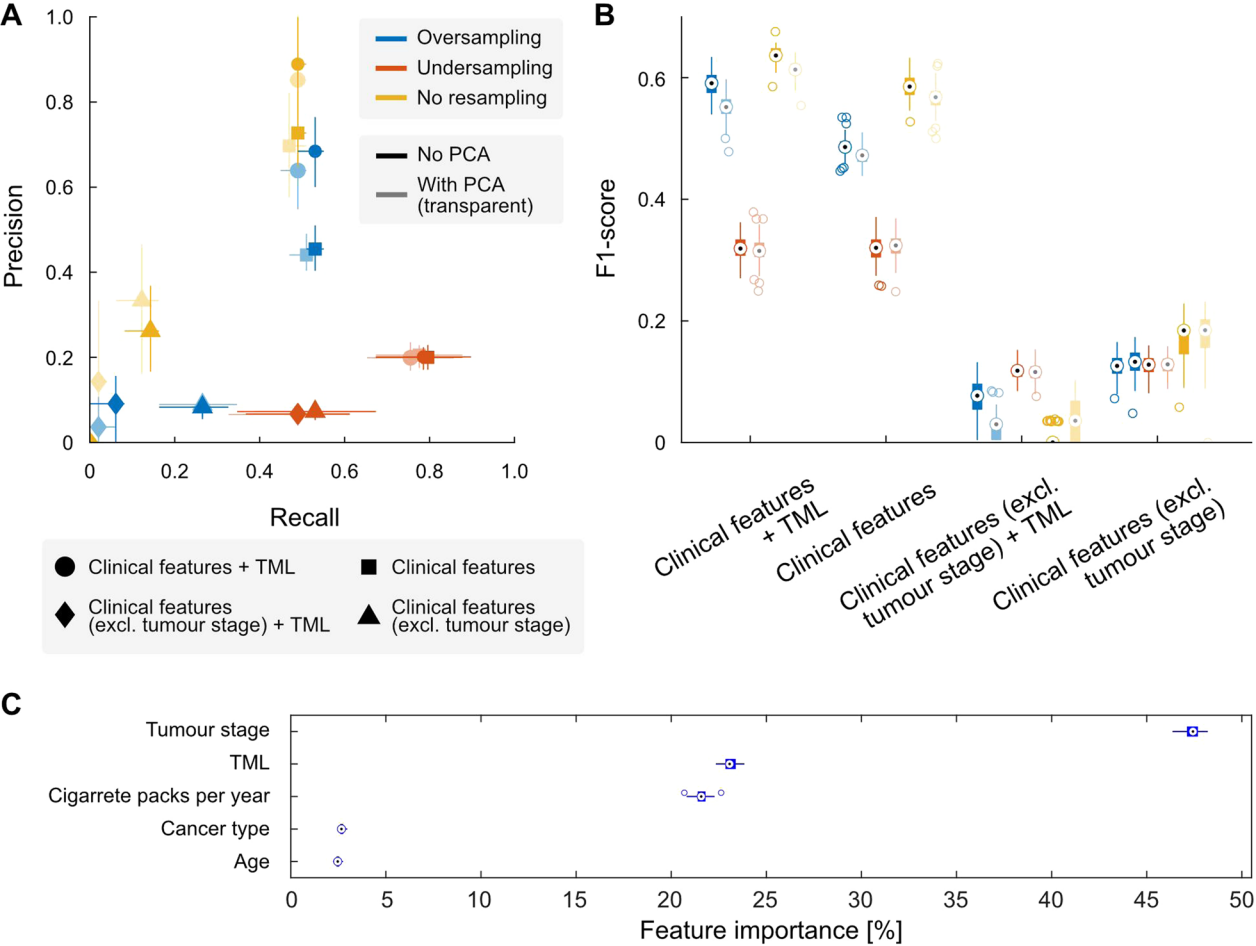
Random Forest models assessment. (**A**) Precision and Recall for 100 realisations of the three RF models, with their 95% confidence intervals and medians. Marker shape accounts for the model evaluated (i.e., trained using these features and subsets thereof), while colours represent the resampling method used. Line opacity stands for whether the data was subject to a PCA (dark lines) or not (transparent lines). (**B**) F1 score for 100 realisations of the three RF models, with medians and 95% confidence intervals. (**C**) Differential contribution of TML and clinical features to model's predictive performance (model 1, without PCA or resampling) for the 100 realisations, with medians and 95% confidence intervals

We compared the performance metrics reached by RF classification models for the RM0 (localised cancer) and RM1 (metastasis) classes. In Fig. 5A, we show all models' Precision and Recall considering the preprocessing and validation methods applied. While predictions for RM0 were accurate, those for RM1 were poor and erratic. We used the Precision and Recall metrics to select the best model, selecting those with the highest values, i.e., in the upper right corner of Fig. 5A.

Models combining clinical variables and TML achieved the highest performance metrics. Moreover, these models applied to datasets without PCA perform better in Recall and Precision, except when metrics are substantially low (cf. Figure 5A). Models trained using both TML and clinical variables (including tumour stage), without using PCA nor resampling, were the best-performing ones (Fig. 5A, B) with an F1 value of 0.64 (95%CrI [0.62, 0.66]). We summarise performance metrics for all other models in Table 3. While clinical variables, in general, seem to be good predictors of metastasic status, the tumour stage is the decisive component to obtain these results (Fig. 5C).

**Table 3:** F1 score of Random Forest classification models

	No PCA			PCA		
	Oversample	Undersample	No resample	Oversample	Undersample	No resample
Clinical Features + TML	0.59 [0.55, 0.63]	0.32 [0.28, 0.36]	0.64 [0.62, 0.66]	0.55 [0.51, 0.59]	0.32 [0.27, 0.37]	0.61 [0.58, 0.64]
Clinical Features	0.49 [0.45, 0.52]	0.32 [0.27, 0.36]	0.59 [0.56, 0.63]	0.47 [0.44, 0.51]	0.32 [0.28, 0.36]	0.57 [0.52, 0.61]
Clinical Features (excl. tumour stage) + TML	0.07 [0.00, 0.11]	0.12 [0.09, 0.14]	0.00 [0.00, 0.04]	0.03 [0.00, 0.08]	0.12 [0.09, 0.14]	0.04 [0.00, 0.07]
Clinical Features (excl. tumour stage)	0.13 [0.08, 0.16]	0.13 [0.09, 0.16]	0.18 [0.11, 0.21]	0.13 [0.09, 0.17]	0.13 [0.09, 0.16]	0.18 [0.09, 0.23]

## Discussion

In this study, we first analysed the association between the number of missense mutations, i.e., the Total Mutational Load (TML), and clinical variables in a cohort of 1144 LUAD and LSCC patients. Then, we used these results to understand the metastasis status classification models using a benchmarking strategy based on Random Forest (RF) models.

Regarding clinical parameters and TML, we found that age, smoking history, and cancer type are significantly associated with the TML. In other words, patients belonging to different categories in these clinical variables had significantly different mean TMLs. Younger patients (≤ 60 years old) showed a higher TML than older patients (> 60 years old), while lifelong non-smokers had lower TMLs than current smokers. When having a closer look, we find among LUAD patients that young current smokers have higher TMLs than old current smokers (cf. Figure 2C, D). Thus, we analysed the dataset to assess whether younger patients in our cohort were heavier smokers than older ones. We found no significant difference between the number of packs of cigarettes smoked per year between age groups in LUAD patients. However, for LSCC patients, older patients have a higher consumption of cigarettes (p = 0.0296) (Additional file 1: Fig. S1). The above suggests that there might be other variables than solely smoking habits and the number of cigarettes necessary to explain this difference.

Regarding smoking status, reformed smokers have higher TML than lifelong non-smokers, suggesting that cigarette consumption has long-term effects on missense mutations. Considering cancer type, LUAD patients have a higher TML than LSCC patients. We found that smoking is strongly associated with the TML in LUAD, consistent with the literature [10]. LUAD never-smoker patients have a lower TML than those who have actively smoked during their lifetime. Therefore, smoking seems to be a relevant factor in explaining the increase in mutations in patients with this disease. On the contrary, LSCC patients that have never smoked have a larger number of missense mutations than never-smoker LUAD patients, indicating that other factors contribute most to the development of this pathology (cf. Figure 2C, D). Consistently, previous studies showed that LSCC patients accumulate numerous passenger mutations and suggested that LSCC is no longer a smoker’s-only disease since 14.7% (95% CI, 12.1% –17.4%) of their patients were never-smokers [22].

Interestingly, the association between TML and smoking status which we found finds support along the lines of recent experimental findings linking smoking with an increased risk of LC and a higher frequency of somatic mutations [23]. This association was also suggested by previous preliminar studies [24]. However, given the observational nature of our study, our results could also be explained by the following confounders. First, we found that the TML was higher in younger than older patients, which could account for different health-seeking behaviours between age categories. For example, as young individuals do not perceive themselves at risk of developing cancer, they do not go through screening until presenting symptoms—which typically appear in advanced stages of cancer. As the TML accounts for how much an individual has been exposed to mutagenetic factors (own and external), it is reasonable to expect specific relation between tumours and accumulation of mutations (deletion of tumour suppressing genes or incorporation of proto-onco genes). However, when combined with the explicit label given by clinical categorization, we found the TML to add information that was not contained in such, confirming the non-redundancy of the variable we put forward. Factors as socioeconomic status, access to health, and typical comorbidities should also be homogeneous across the cohort and could explain potential differences with other studies. Given the high costs related to tumour typification, economic status and whether the governments mediate access to health are determinants for correct classification within the cohort and can further increase class imbalance in cohorts from countries with lower average incomes.

Understandably, there are several ways to quantify the effects of mutational processes. In our case, we counted all missense mutations in the tumour exome to define the TML. Although this modelling choice can be considered somewhat arbitrary, we do not aim to provide a general tool for characterizing mutagenesis nor compare alternatives for that, but rather to generate a method that answers our research questions. In that way, our study opens new research directions to assess whether other calculation methodologies for the TML could further improve the classification performance of RF models trained with it.

Although we have a large cohort of patients, the main difficulty in this work (and related works) is the imbalance of classes in the metastatic status. Notwithstanding the technical challenges behind dealing with class imbalance, we found that models trained on datasets without PCA and no data resampling achieved the best classification performance. Regarding the PCA, obtaining models with low performance might be due to information losses induced by the reduction in dimensionality. Regarding resampling, not performing any led to higher performances, but might also lead to overfitting, especially when using low values of k for the k cross-validation. Finally, we verified that models trained using clinical variables and TML obtained the best performance metrics. The tumour stage, redefined including also early stages as described in Methods, is a very relevant variable in the classification model. These results indicate that tumour stage II and III samples could be reclassified as metastatic samples being able to help the pathologist to classify samples considering this information. On the other hand, TML is also ranked as a highly-informative variable, suggesting that the information contained on it is not redundant with clinical variables.

Altogether, the findings in this work may contribute to the development of diagnostic tools able to classify metastasis status at an early stage using clinical information, such as the cancer type, the smoking history, and the age. For example, we knew that smoking has a critical relationship with the generation of LC. However, according to our results and when combining both variables, TML has a more important contribution to predicting metastasis in patients with this disease than cigarette smoking. Therefore, we remark on the benefits of including it as a predictive feature in classification models driven by machine learning.

## Conclusions

We demonstrate that using clinical variables, such as cancer type, smoking status and frequency, together with TML, allows predicting whether a patient with LUAD or LSCC will develop metastasis with higher certainty. Altogether, we contribute to developing more effective and personalised molecular tools for tumour typification and cancer diagnosis. Thereby, we provide practitioners with more alternatives to promptly identify the best treatment to increase the life expectancy of their patients.

## Supplementary Information

**Additional file 1:** **Figure S1. **Association between cigarette consumption and age in LUAD and LSCC patients. Although the cigarette consumption distributions have different widths, being broader for younger patients, there is only a weak difference between median values. For LUAD patients, no difference was found with a p-value just above the significance threshold. For LSCC patients the situation is the opposite, supported by a p-value just below the significance threshold.

## Data Availability

We analysed public data from CbioPortal for LUAD and LSCC cancers (https://www.cbioportal.org/). Analysis codes are available at a GitHub repository (https://github.com/StarBrand/rf-tml). This manuscript is part of Dr. Karen Oróstica PhD thesis work at Universidad de Chile and is publicly available at the institutional repository.
