# Supplementary material for: Total mutational load and clinical features as predictors of the metastatic status in lung adenocarcinoma and squamous cell carcinoma patients
Source: J Transl Med. 2022 Aug 18;20:373. doi: 10.1186/s12967-022-03572-8 (PMC9389677; doi:10.1186/s12967-022-03572-8)
Supplement: Supplementary file 1 — Additional file 1: Figure S1. Association between cigarette consumption and age in LUAD and LSCC patients. Although the cigarette consumption distributions have different widths, being broader for younger patients, there is only a weak difference between median values. For LUAD patients, no difference was found with a p-value just above the significance threshold. For LSCC patients the situation is the opposite, supported by a p-value just below the significance threshold. [file 12967_2022_3572_MOESM1_ESM.docx]

**Additional file Figures**


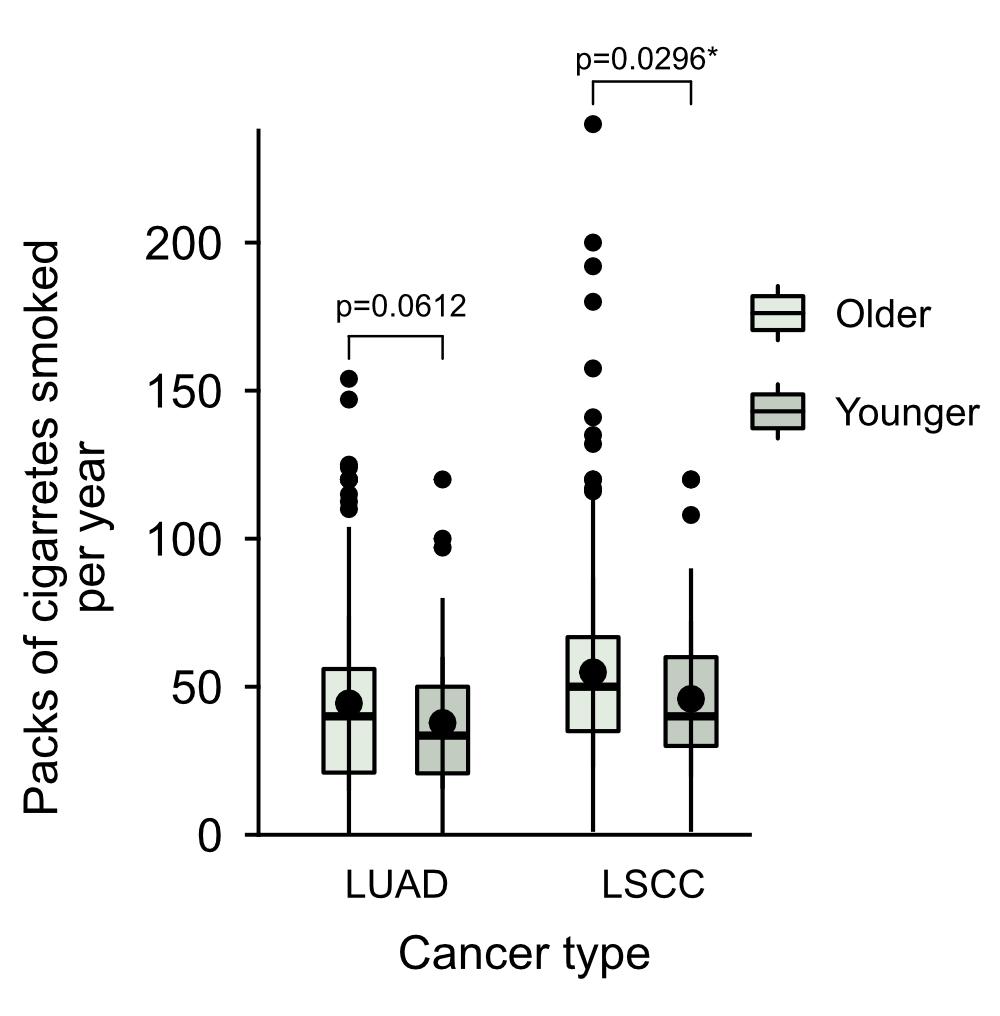


Additional Figure S1: **Association between cigarette consumption and age in LUAD and LSCC patients**. Although the cigarette consumption distributions have different widths, being broader for younger patients, there is only a weak difference between median values. For LUAD patients, no difference was found with a p-value just above the significance threshold. For LSCC patients the situation is the opposite, supported by a p-value just below the significance threshold.
